# Developing Biostimulants From Agro-Food and Industrial By-Products

**DOI:** 10.3389/fpls.2018.01567

**Published:** 2018-10-30

**Authors:** Lin Xu, Danny Geelen

**Affiliations:** Horticell, Department of Plant and Crops, Faculty of Bioscience Engineering, Ghent University, Ghent, Belgium

**Keywords:** biofertilizer, agrochemical, organic waste stream, bio-economy, sustainable farming, plant extract

## Abstract

In modern agriculture, seeking eco-friendly ways to promote plant growth and enhance crop productivity is of priority. Biostimulants are a group of substances from natural origin that contribute to boosting plant yield and nutrient uptake, while reducing the dependency on chemical fertilizers. Developing biostimulants from by-products paves the path to waste recycling and reduction, generating benefits for growers, food industry, registration and distribution companies, as well as consumers. The criteria to select designated by-products for valorizing as biostimulant are: absence of pesticide residue, low cost of collection and storage, sufficient supply and synergy with other valorization paths. Over the years, projects on national and international levels such as NOSHAN, SUNNIVA, and Bio2Bio have been initiated (i) to explore valorization of by-products for food and agriculture industries; (ii) to investigate mode of action of biostimulants from organic waste streams. Several classes of waste-derived biostimulants or raw organic material with biostimulant components were shown to be effective in agriculture and horticulture, including vermicompost, composted urban waste, sewage sludge, protein hydrolysate, and chitin/chitosan derivatives. As the global market for biostimulants continues to rise, it is expected that more research and development will expand the list of biostimulants from by-products. Global nutrient imbalance also requires biostimulant to be developed for targeted market. Here, we review examples of biostimulants derived from agricultural by-products and discuss why agricultural biomass is a particularly valuable source for the development of new agrochemical products.

## Introduction

Due to increasing demand for better yield and quality of food and crops, seeking eco-friendly and sustainable ways to produce fertilization reagents of biological origins has become a major goal in agriculture. Biostimulants are products able to act on plants’ metabolic and enzymatic processes improving productivity and crop quality. It also assists plants to cope with abiotic stress, especially in the early stage of plant development. European Biostimulants Industry Council (EBIC) defines biostimulants as “substance(s) and/or micro-organisms whose function when applied to plants or the rhizosphere is to stimulate natural processes to enhance/benefit nutrient uptake, nutrient efficiency, tolerance to abiotic stress, and crop quality^1^.” The economic relevance of such products is not insignificant as the global market for biostimulants has been projected to reach $ 2,241 million by 2018, having a compound annual growth rate of 12.5% from 2013 to 2018 (Calvo et al., 2014). In the European Union, the economic value of biostimulants is estimated to be between 200 and 400 million euros (with a yearly growth of 10%) (EBIC). Owing to the positive effects of biostimulant on plant growth, reduction of stress and disease prevention, the use of biostimulant contributes to boosting plant production, yield and quality.

Over the years, several types of biostimulants have been defined by different authors, based on source material, mode of action and other parameters [Reviewed in (Yakhin et al., 2016)]. For example, du Jardin (2015) categorized biostimulants into 7 classes: humic acid (HA) and fulvic acid (FA), protein hydrolysates (PHs), seaweed extracts, chitosan, inorganic compounds, beneficial fungi and bacteria (du Jardin, 2015). The primary sources of biostimulants also display various origins and physiological characteristics. It reflects on the categorization of biostimulants as well. For example, macroalgae and their extracts have long been used for biostimulant production (McHugh, 2013).

The active ingredients present in different types of biomass with potential biostimulant activity fall into a diverse range of molecules that includes phytohormones (cytokinin, auxins, gibberellins, brassinosteriods, ethylene, and abscisic acids) (Letham and Palni, 1983; Mussig et al., 2006; Pimenta Lange and Lange, 2006; Werner and Schmulling, 2009; Wolters and Jurgens, 2009; Zhao, 2010; Pacifici et al., 2015), amino acids (Hoque et al., 2007; Colla et al., 2015; Colla et al., 2017), polyamine (Fuell et al., 2010), etc. In seaweed extracts, phytohormones have been demonstrated to be present and are considered as putative bioactive ingredients of this category of biostimulants (Khan et al., 2009; Stirk et al., 2014; Stirk and Van Staden, 2014). In addition to hormones, algal extracts also contain a range of carbohydrates such as alginate, fucoidan, betaines as well as proteins and minerals which promote plant growth (Sharma et al., 2014).

It should be noted that waste streams from food and agricultural industries are equally important sources for biostimulant development. The biostimulants generated from waste streams are extracts from food waste, composts, manures, vermicompost, aquaculture waste streams and sewage treatments (Yakhin et al., 2016). PHs are commonly used biostimulants, which are a group of polypeptides, oligopeptides, and amino acids that are manufactured from hydrolyzed protein-rich waste (Schaafsma, 2009).

Recycling of organic waste as biofertilizers is historically a common practice. Manure has been and is applied to the field as soil fertilizers across the world. However, environmental problems occur when the local production exceeds storage and local application capacity, resulting in careless disposal and associated environmental spillage with consequent water pollution, over-fertilization, ammonia toxicity, and infestation of human pathogens, etc. (Dominguez and Edwards, 2010). To avoid environmental contamination, finding alternative ways to reuse such material in the agricultural production system is necessary. The concept of circular economy emphasizes the process of converting waste materials and products that have reached the end of their life cycle into new sources. A new EU regulation that entered into force on January 1, 2018 aims at boosting the use of bio-base wastes as new types of fertilizers (European Commission, 2016a). These waste-originated fertilizers usually contain biostimulatory substances, and we will discuss further in this review when placing composts as important sources of biostimulants. When a waste turns into raw material for industrial or agricultural application, then it is no longer waste. As the definition of “waste” describes the material to be discarded, it is sometimes more appropriate to label the material as by-product. A by-product is lawfully used, not deliberately produced, of certain use, ready for use without further processing and produced as an integral part of the production process (European Commission, 2007).

Through research and development of using industrial waste for re-manufacturing, reuse and recycling, one can make fundamental steps toward biomass optimization and resources use efficiency and sustainability (European Commission, 2017). Therefore, developing biostimulants from by-products provides the innovative methods to prevent inadvertent disposal and results in environmental-friendly solutions for waste re-use. From a legal point of view, the European Commission is proposing a Regulation which will ease the access of organic and waste-based fertilizers to the EU market (European Commission, 2016b). In this review, we give a current view of strategies employed to turn organic wastes into added-value products, especially in the field of biostimulant development. A number of categories of biostimulants is presented, providing a glimpse of biostimulant products currently on the market.

## Defining Biostimulant Derived From Waste Stream

### By-Products and Their Utilization in Food and Feed

With the demand for larger quantities of healthy and fresh food, the agricultural and food industry companies are faced with tremendous amount of organic biomass, coming from the production or processing procedures. Depending on the types of crop and its processing, the scale of biomass production may substantially vary. For example, fish by-product consists of over 60% of the biomass, including head, skin, fins, frames, etc. (Chalamaiah et al., 2012). The fishing industry produces vast amounts of the exoskeletons of crustaceans, coming from shrimp, crab and lobster, mounting to a global production of 5.9 Mt, with 35–45% of it being discarded as waste (head and thorax) (Sharp, 2013). A lot of these sources are enriched in secondary metabolites because they originate from cells and tissues exposed to the exterior of the organisms’ body which is developed to keep off attackers and pathogens. For instance, potato peel is enriched in steroidal alkaloids which are associated with defense against bacterial, fungal, and insect pathogens [Reviewed in (Fritsch et al., 2017)]. In tanning industry, which treats animal skins and hides to produce leather, it generates large quantities of by-products: one metric ton of wet salted hides produces 200 kg of leather and 450–600 kg solid waste, leaving more than 60% of biomass, if not converted, will be disposed to the environment as waste (Alexander et al., 1992; Verheijen et al., 1996). Thus, the sheer volume of by-product is vast and the presence of a complex mix of metabolites profile is favoring the exploitation of these products.

Coming to terming with the problem of disposing or re-using industrial by-products, techniques have been developed to process fish by-products, such as enzymatic hydrolysis, autolysis and thermal hydrolysis (Halim et al., 2016). The human health benefits associated with fish PHs (FPHs) are demonstrated by the commercial preparations used as healthy food and/or nutraceuticals in many countries (Chalamaiah et al., 2012). The antioxidant properties of FPHs are associated with protecting the human body from oxidative stress (Chalamaiah et al., 2012). Other positive effects on human physiology are attributed to fish by-product as well, including the prevention of high blood pressure (hypertension), as well as anti-cancer activity (Halim et al., 2016). Besides development of human consumption, by-products are also being used as animal feed. FPHs have been used in aquaculture feed to enhance the growth and survival of fish (Kotzamanis et al., 2007).

Another waste-turn-into-feed example comes from feather waste that is enriched in keratin, a nutrient source for animals and plants (Korniłłowicz-Kowalska and Bohacz, 2011). Chicken feathers are the most common keratin waste product with high amounts being produced in poultry slaughterhouses (Korniłłowicz-Kowalska and Bohacz, 2011). Owing to its high protein content, feather waste has been largely converted to feedstock. However, the application of animal by-product used as feedstock is declining over the years because of more stringent regulations. Besides, valorization strategies of animal feed come with limited financial return. On the other hand, PHs are now accepted as components of plant biostimulants in agriculture that increase productivity and quality of crops (Colla et al., 2017). It should be noted that animal by-products not intended for human consumption are potential sources of risks to the public and to animals. An example of the problems that may arise because of recycling animal waste is the use of certain animal by-products that gave rise to outbreaks of foot-and-mouth disease, bovine spongiform encephalopathy (BSE) (European Commission, 2009).

### Suitability of Waste Streams Used for Biostimulant Development

As the first step toward developing biostimulants from organic waste, the choice of biomass resource is critical. Various active ingredients found in industrial waste streams and by-products of biological origins pose the perfect opportunity to extract molecules for better growth and pathogen resistance of valuable crop plants. However, some understanding of the intrinsic biochemical characteristics of raw materials is needed, such as preserving the specific bioactive ingredients (Povero et al., 2016). The environmental and economic evaluation must be carried out as well, to assess whether a new biostimulant product has good prospect to become successful. To assess if the raw material is suitable for the development of biostimulants, several factors must be taken into consideration.

#### Absence of Pesticides

It is expected that during conventional pest management, pesticides have been used to safeguard crop production. By-product derived from plant species that has been treated with pesticides could potentially cause problems for biostimulants production, as it no longer will be seen as a “natural” product and will be considered as an alternative preparation of the regulated agrochemical. In most EU member states, it is specified that biostimulant-like substances are different from plant protection products and they must not harm the wellbeing of humans, animals and the environment (La Torre et al., 2016). Hence, contamination by pesticides should be prevented with maximal measures when obtaining the source material. Even the smallest amount of contamination with pesticides may cause problems with the legislation. Since during the extraction procedure, solvents (e.g., n-hexane) may selectively solubilize and concentrate pesticides from water matrices (Zayats et al., 2013). To this end, the source of plant biostimulants should not contain pesticides that otherwise leads to problems with the registration and permission to use in sustainable agriculture.

#### Low Cost of Collection and Storage

The initial economic value of the waste material should be low and it preferably requires additional processing to dispose of at a financial cost. The reason for this is that the yield increase attributed to biostimulant application is typically in the range of 5–10% and this limits the profitability of biostimulant sales. The economic burden of a by-product resource can be broken down into three main components: collection, conservation and storage, and transport. Taking the example of fish by-products, the intrinsic value for the production of food and animal feed, coupled with the high cost to preserve fish biomass at low temperature, all lead to the recovery of by-products for further valorization unprofitable. To achieve the recovery of by-product, some fish species are often brought ashore for processing, in order to overcome limited preservation space of by-product while at sea [reviewed in (Olsen et al., 2014)]. Despite the complexity of organizing the logistic of storage for perishable biomass, agricultural production is typically linked with seasonal production activities and poor conservation methods may in addition result in deterioration of the material that could affect the stability of bioactive ingredients. Many natural products are unstable and undergo chemical modification when exposed to heat, light or oxygen, and result in loss of bioactivity (Turek and Stintzing, 2013). A sound business model that addresses the logistic problems of collection and storage of resources is more likely to become successful if it is derived from a stable source material.

#### Sufficient Availability

An effective agrochemical product is preferably available in sufficient quantities to accommodate the market demand. It is therefore critical that the biomass from which a biostimulant product is derived is available in large quantities and readily available for processing. A good example of an abundant waste product, yet highly perishable, is the heads and shells of crustaceans that are a source of chitin. It should also be avoided to develop biostimulants derived from by-products which are prone to perturbation, i.e., the material that strongly varies in composition or for which the supply is uncertain. An abundant product has, in addition, the advantage of attracting further valorization and development for other usage. The ample quantity and diversified applications can be demonstrated in the case of seaweed. As an essential source of biostimulants with already commercialized products, the seaweed industry provides an annual input of 7.5–8 million tons of wet seaweed, which is used for food, fertilizers, feed, biofuel, and cosmetics, etc. (McHugh, 2013). Among them, brown algae are the groups of seaweed having large tonnage whose extracts are exploited widely as a source of biostimulants. For sewage sludge, less exploited but with great potential for nutrient recovery, the annual flux is around 9.5 million tons in EU member states (Buckwell and Nadeu, 2016). Taken these issues into consideration, it is advised that a careful selection of materials is necessary for generating new types of biostimulant: from processing, extraction, formulation to marketing and distribution.

#### Positive and Negative Impact of Competing Use: A Double-Edged Sword

If different valorization strategies applied to the same waste stream are in competition with each other, which may turn out to have a negative impact on the development of a biostimulant product. This is because biostimulants are typically less valuable than animal feed and much less than valorization through the isolation of fine chemicals as pharmaceutical agents. Consequently, to be suitable as biostimulant source, the waste source is preferably not intensively used for feed or other higher value uses. An overview of the biomass value pyramid has been presented by Meyer (2017) in the context of developing bio-based economy (Meyer, 2017). Although there’s no clear prioritization of different sectors of biomass use, it is most valuable when biomass is used as pharmaceuticals or fine chemicals to benefit health and lifestyle (Meyer, 2017). The waste-derived biostimulants currently on the market originate from only a few sources (e.g., plant- and animal-derived PHs). Further valorization through development of biostimulants from the same sources may be difficult due to intellectual property issues.

However, on the other hand, when a biomass is being used for other purposes, development of a new biostimulant from that source remains an option. To avoid the competition, it requires high compatibility of the two processing technologies, and when integration is feasible, it may even generate greater benefit. Such synergistic situations are currently still rare and further maturing of the biostimulant research is likely to generate opportunities for process integration. This is for example the case for various seaweeds that are now well accepted to harbor biostimulatory activity, as dozens of manufacturers around the world have launched formulated seaweed products specifically tailored for various crops (Sharma et al., 2014). In olive oil industry, 95% of olive by-product are currently consumed as low-value uses (energy generation, composting), with the remaining 5% as animal feed (Berbel and Posadillo, 2018). However, it doesn’t hamper the processing development of olive biomass for high-value uses such as extraction of bioactive compounds (i.e., phenol) (Berbel and Posadillo, 2018). Consequently, there are both positive and negative aspects to consider when a given biomass is used in diversified sectors. Researchers and entrepreneurs are suggested to carry out a SWOT analysis, identifying the pros and cons when developing biostimulants from material that is being exploited for different purposes already.

## Valorization of Organic Wastes: Examples of Cross-Regional Funded Projects

Valorization is the process of converting by-products to high value substances. The development of novel tools to secure crop yield are objectives that are of high importance on the agenda of the research funding bodies across Europe and worldwide. On the other hand, increasing price of oil directly affects the cost of fertilizers, which urges farmers to look for novel ways to reduce the input cost. The concept of circular economy creates opportunities for the agricultural and food sector aiming at valorizing by-products. Indeed, recycling raw organic material from waste is a first step toward reducing energy and material input cost in the production process. Besides feedstocks, by-products can be converted to bio-based chemicals, biostimulants and soil amendments, as well as value-added products in biomaterial industries (e.g., bioplastic, lignin and alginate) (De Corato et al., 2018).

**FIGURE 1:**
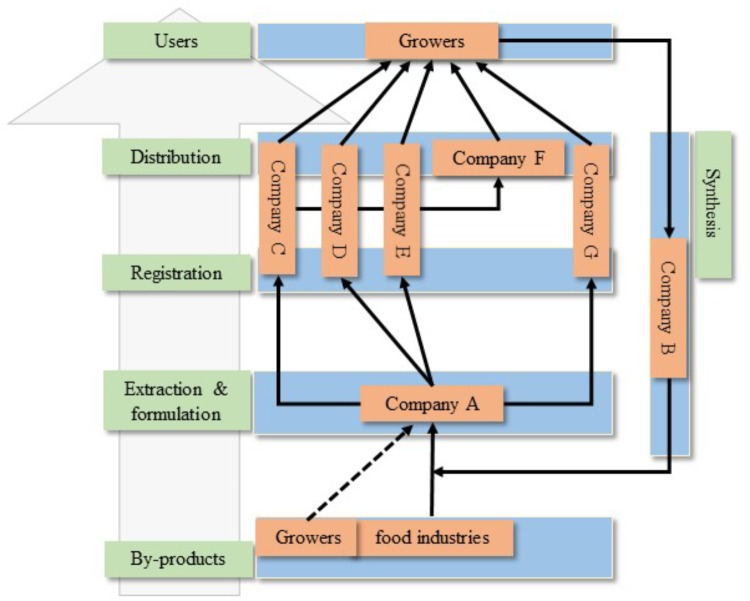
Possible scenario in valorization chain of biostimulants from waste streams. Food industries and growers generate substantial amount of waste material and they are seeking methods to valorize as it costs to process the by-products. Food industries have the capacity to transport the by-product out of sites. Growers, however, usually lack the logistic means to process the by-products. Thus, a limiting factor for growers to valorize the crop residues is the cost of transport. As the first step toward valorization, Company a has the expertise in extraction and formulation from by-products. Company C–E, and G are heavily involved in the production and marketing of biostimulant products and they invest in registration and distribution. In spite of the ability to produce bioactive ingredients, these companies might still need assistance from Company a in extraction and formulation. Company C–E are also selling biostimulants to intermediate companies (Company F), who produce seeds, substrates or fertilizers. It is likely that one new biostimulant will be marketed as substrate, soil improvers or seed-coating. The ultimate target groups of biostimulants are crop growers. The proved efficacy of one biostimulant from growers will inspire the identification of chemical structure by Company B, carrying out synthesis of analogs. Consequently, it provides further knowledge into the valorization chain since the extraction and formulation can be designed to isolate the targeted bioactive ingredients.

To promote the research and development toward valorizing organic wastes, a few funded projects are published within European Commission. The NOSHAN Project (Functional and Safe Feed from Food Waste, EU FP7 Grant No. 312140) investigated food waste processing and technologies to use for feed production at low cost. Food waste has good nutritional value provided it is treated and conserved correctly^2^. An example is pectin that has been characterized in 26 different food waste streams. The NOSHAN project revealed that the pectin structures and yield are highly diverse (Muller-Maatsch et al., 2016). Furthermore, the study provides insight into using pectin as food additive from a variety of waste source material (Muller-Maatsch et al., 2016). SUNNIVA focused on reducing waste by providing valorization strategies in vegetable processing^3^. It envisioned optimizing strategies to exploit by-product from food production and develop fertilizers from waste (field and storage) and processing side-flow. For instance, vegetable waste and side-flow were assessed for their use as organic fertilizers. The raw material fractions were tested to identify their effects on plant growth and defense and their suitability to host beneficial microorganisms. Within Bio-Based Industries Joint Undertaking (BBI-JU), operating under H2020 Framework Projects, one of the key areas is to find solutions for waste reduction and strategies to valorize waste. In this regard, AgriMax^4^, FUNGUSCHAIN^5^, and NEWFERT^6^ focus on recovering organic waste, filling the gaps of nutrient cycle for the food and fertilizer industries. It is postulated that waste and by-products from potato, tomato, cereals and olive have great potential to be valorized on the EU market as food additives, agricultural materials, packaging materials and biofertilizers (Fritsch et al., 2017). Figure 1 illustrates the valorization chain starting from the suppliers of waste material to end users, illustrating the economic potential of biostimulants from waste streams.

## Tackling Modes of Action of Biostimulants From Organic Waste

“Mode of action” implies the detailed biochemical and physiological changes of the plants upon application of biostimulants. Despite the strong interest in developing new biostimulants, few well-characterized products with reliable performance are on the market. Generally there is limited insight into the mode of action, largely due to the diversity of source material and the complexity of the resulting product which is typically complex and chemically poorly characterized (Brown and Saa, 2015). Especially when material comes from living microbial cultures, macro and micro-algae, PHs, vermicompost and other types of industrial wastes, complexity is extremely high and multiple components are likely to be involved (Brown and Saa, 2015). Despite the complexity of the substance mixture, attempts to characterize bioactive ingredients are highly relevant to gain trust and reliability of the product marketed. Furthermore, subsequent stages of action in the plants after application also need to be taken into account: penetration into tissues, reaction with plant metabolites and chemical stability, binding to metabolic enzymes and impact of the compound on the plant’s physiology and modulation of gene expression (Yakhin et al., 2016). It requires a systematic and multidisciplinary approach whereby various technologies in the fields of chemistry, biology and genomics need to be combined (Povero et al., 2016).

The investigation of the mode of action of a biostimulant requires two consecutive steps: determination of active ingredient(s) and its/their mode of action on plants. Omics approaches are routinely employed in modern life science and they involve high-throughput technologies that are capable of measuring global changes in the abundance of mRNA transcripts (transcriptome), proteome, and metabolome in complex biological systems as a result of biochemical stimulations or perturbation. They provide reliable and informative tools to unveil the mode of action of biostimulants and hence mode-of-action studies rely heavily on omics tools. A transcriptomic study revealed that HS extracted from vermicompost modified plant physiology and metabolism by regulating expression levels of genes involved in cell cycle and meristem and cytoskeleton organization (Trevisan et al., 2011). Similarly, proteomic data showed that chitosan modulates protein abundance belonging to primary metabolism pathways in grape (Ferri et al., 2014). We have recently embarked on a project with the objective to identify biostimulants and biopesticides from agricultural waste streams ^7^. The project “Bio2Bio” investigates material from organic waste streams and by-products from food industries in Flanders, creating a library of formulated extracts. With the help of a bioassay screening platform, promising extracts and fractions are being characterized. The screening platform gathers the expertise from a variety of *in vitro*, greenhouse and field tests to score the materials for biostimulatory activity. Most importantly, Bio2Bio aims to determine bio-active ingredients and studies their mode of action. The impact of the project lies in the discovery of potentially new bioactive compounds of natural origin. The new ideas from the research part of the project will provide valuable leads for the agrochemical companies to develop new biostimulant products, benefiting the different target groups. The valorization potential can also be benefited from the mode of action studies.

## Current Examples of Biostimulants From Organic Waste and By-Products

One of the important valorization strategies for by-products is to exploit bioactive compounds to improve plant growth and resistance to pathogens. Several classes of organic wastes or by-products that are currently valorized as plant biostimulants, which are claimed to render better plant growth and increased pest tolerance. In this section, we are introducing the biostimulant substances processed or extracted from different classes of source material that are considered to be wastes and by-products in the agriculture and food sectors, including substances from composting, PH, chitin and chitosan, and other by-products from biological origins. According to EBIC definition, compost doesn’t fall into the category of the biostimulants and adheres better to biofertilizers. However, compost is a source for agrochemicals or microbes that potentially display biostimulant properties, such as HS, phytohormones, amino acids and other substances that can be extracted from it. These substances function by interacting with plant signaling processes and reducing negative response to stress, rather than by virtue of these substances (Brown and Saa, 2015). Typical biostimulant effects have been ascribed to composts, including enhancement of nutrient uptake, improved pathogen defense, etc. Moreover, the application methods, whether foliar spray or adding to soil, do not determine whether a product display biostimulatory activity or not. To this end, composted material exemplifies waste-derived sources of biostimulants, as various biostimulant-related studies have utilized active ingredients from compost to promote plant growth and protect against disease. In addition, composting is conforming to the concept of circular bio-economy.

### Biostimulants From Vermicompost

Vermicompost is the organic matter processed by earthworms. The techniques of vermicomposting have been widely applied to reduce the volume of plant organic waste, manure, paper, food and sewage sludge (Dominguez and Edwards, 2010; Allardice et al., 2015). Vermicomposting helps to eliminate the occurrence of human pathogens present in manure, including fecal coliforms, *Salmonella* species, enteric viruses and helminthes (Eastman et al., 2001; Edwards and Subler, 2010). It can also be used as a substitute for peat, which is usually mined in unique wetland ecosystems, in potting media with biostimulatory effects on seedling performance, altering fruit quality (Zaller, 2007). As a result, the process of vermicomposting brings about more sustainable waste management strategies that may otherwise pose health risks, contaminating our living environment (Eastman et al., 2001; Edwards and Subler, 2010).

Biostimulants can be extracted from vermicompost, which are utilized in plant growth media and soil amendment, alleviating of nutrient deficiency and abiotic stress (Aremu et al., 2012; Chinsamy et al., 2013; Aremu et al., 2014). The biostimulatory effects of vermicompost are attributed to the presence of substances with phytohormonal activity. A mixture of plant growth regulators (PGRs), cytokinins, auxins, abscisic acid, gibberellins, and brassinosteroids were shown to occur in leachate of commercially produced vermicomposted garden waste (Aremu et al., 2015). Also HA and FA are present and are potentially responsible for the biostimulant activities of vermicompost. The composting process provides more stable and mature organic material, and in term results in enrichment of humic substances (HS) (Provenzano et al., 2001). The direct effect of HS on plant growth is suggested to come from its interaction with the plant membrane transporters. This supposedly increases nutrient uptake and trigger membrane-associated signal transduction cascades which regulate growth and development (Canellas et al., 2015). Most reported beneficial effects of HS correlate with changes in root architecture. HS enhances the H^+^ efflux activity in the elongation and differentiation root zone which has a stimulatory effect on nutrient uptake and lateral root emergence and root hair [Reviewed in (Canellas et al., 2015)]. In maize (*Zea mays* L.), a single application of HS, which is extracted from vermicomposted cattle manure, decreases the leaf total carbohydrate content, fructose and glucose contents, while increases starch content, indicating a role in N and C metabolism (Canellas et al., 2013). The application of HS isolated from vermicompost, in combination with diazotrophic endophytic bacterial inoculation was shown to strongly promote plant growth (Canellas et al., 2015).

There are several beneficial implications of vermicomposting in sustainable agriculture and nutrient recycling. Vermicomposting is a cost-effective and sustainable waste management tool that can be readily scaled up (Pathma and Sakthivel, 2012; Yadav and Garg, 2013). It circumvents many of the challenges associated with handling raw organic wastes (Dominguez and Edwards, 2010). Importantly, vermicomposting allows growers to recycle wastes from their own farming activities (e.g., plant materials and animal manure), as well as turning suitable wastes into effective organic fertilizers that improve crop production yield, while improving soil fertility at the same time (Brown et al., 2004; Laossi et al., 2010; Van Groenigen et al., 2014).

### Biostimulants From Other Types of Compost

Composting of organic material is the aerobic degradation through a series of microbial reactions transforming the organic material to molecules of smaller size. Suitable as a source of plant biostimulant, composted municipal solid waste are useful for HA extraction (Jindo et al., 2012). When amended with bulking agents, it improves the growth of ornamental plants (Zhang et al., 2013; Zhang et al., 2014). Compost of municipal organic waste was also shown to improve Fe uptake of pear trees and augmented fruit quality (i.e., size, soluble solid concentration) (Sorrenti et al., 2012). In another report, the small molecule size of the composted garden waste was proposed to allow faster uptake by the tomato plants and improved the productivity (Sortino et al., 2014). Depending on the anaerobic or aerobic pre-treatment, urban organic waste is a source of different products (Massa et al., 2016). It is however recommended that such material be tailored to address the needs of specific plant species or cultivation conditions. The composition of bioactive ingredients varies in different composted organic waste, as HS extracted from composts of a number of organic waste sources (i.e., residues from artichoke, funnel, tomato, and cauliflower) were evaluated to harbor different biostimulant capacities (Monda et al., 2018). Most recently, composted olive mill waste water, a by-product from the olive oil industry, is used for HS extraction (Palumbo et al., 2018). Furthermore, although the mechanism remains elusive, the combinatory application of composted organic waste and *Trichoderma* stimulates the level of antioxidant enzyme *in planta* (Bernal-Vicente et al., 2015). This type of synergic biostimulatory capability between microorganisms and compost (or vermicompost) has also been reported previously, such as *Herbaspirillum seropedicae* and HS induced growth and pathogen resistance, possibly by auxin-like and membrane H^+^-ATPase activities (Canellas et al., 2013).

Sewage sludge is the left-over residue generated at centralized wastewater treatment plants (Harrison et al., 2006). Although sewage sludge is used widely in agriculture to recycle mineral nutrients, the presence of heavy metals, organic contaminants and pathogenic bacteria may create risks (Zuloaga et al., 2012). Thus, an EU Directive prohibits the use of untreated sludge for agricultural land applications, and demands the removal of heavy metal and contaminants before using it as biofertilizer (European Commission, 1986). Composting is the main approach to stabilize the sewage sludge for use in sustainable agriculture. Composted sewage sludge (CSS) has been evaluated for the cultivation of vegetable species (Cai et al., 2010). CSS is tested as potting media for lettuce, to replace peat (Jayasinghe, 2012). Biostimulant substances can be found in CSS as well. HS extracted from CSS has been shown to promote root growth and proton pump activity in maize vesicles (Jindo et al., 2012).. In addition, two sanitized sewage sludge streams are used to improve growth and yield of pepper (*Capsicum annuum* L. cv. Piquillo), possibly by promoting rhizosphere microorganism activity and HS (Pascual et al., 2010). As these studies demonstrate the utility of sewage sludge as biofertilizer or source of plant biostimulants, the focus should be on finding solutions for the potential contamination with pathogenic microbes and toxic chemicals, in order to rule out all health risks.

### Protein Hydrolysates

PHs are mainly produced from the chemical and/or enzymatic hydrolysis of proteins from by-products from the agriculture industries, both animal- and plant- origins [reviewed in (Colla et al., 2014)]. The production process and protein source are the major factors determining the chemical properties of PHs (Colla et al., 2015). PHs of animal origin are chemically hydrolyzed by acids and alkalis which increases their salinity (Colla et al., 2015). Considered to be more environmental-friendly, PHs of plant origin are produced by enzymatic hydrolysis, which results in mixture of amino acids and peptides of different lengths with low salinity (Colla et al., 2015). The sources of PHs are diverse, including animal epithelial or connective tissues, animal collagen and elastin, carob germ proteins, alfalfa residue, wheat-condensed distiller solubles, *Nicotiana* cell wall glycoproteins and algal proteins [Reviewed in (Calvo et al., 2014)].

PHs converted from organic waste have been widely promoted as plant biostimulants. Typically, insoluble and soluble fractions are obtained by alkaline hydrolysis. Different material sources are used, such as the remaining biomass from tomato plants at the end of cropping seasons (Baglieri et al., 2014), by-product extracts from apple seeds, rapeseeds, and rice husks (Donno et al., 2013). The enzymatic process to produce amino acids and proteins from carob germs, the by-products of carob fruit, and its positive impact on tomato growth parameters, has also been described (Parrado et al., 2008). PHs from enzymatic extract of vegetable by-products, together with the phytohormone (auxins, gibberellins, cytokinins), improved the anthocyanin levels in grapes (Parrado et al., 2007). Moreover, to avoid heavy metal precipitations, the hydrolysis of sewage sludge has been reported to consist of peptides and free amino acids, and it has potentially biostimulatory effects (Tejada et al., 2013;Tejada et al., 2016). On the other hand, it contributes to alleviating the negative effects on soil enzymatic activities and microbial diversity imposed by herbicide application (Rodríguez-Morgado et al., 2014;Tejada et al., 2014).

When it comes to PHs of animal origin, waste management from the tanning industry turns PHs into a product that was shown to have biostimulants activity (Ertani et al., 2013a;Vaskova et al., 2013). PHs derived from chicken feather result in increased maize yield (Tejada et al., 2018). Siapton, a product from animal PH, acts as an alleviator of salt stress (Mladenova et al., 1998). Another PH with great potential as plant biostimulants is fish PHs. Fish PHs can be derived from fish skin, a rich source of collagen and gelatin (Chalamaiah et al., 2012). Other by-products from the fish processing industries include fish head, muscle, viscera, liver, bone, frame, and roe/egg [Reviewed in (Chalamaiah et al., 2012)]. Fish by-products are enriched in proteins, fat, amino acids (after hydrolysis), antioxidant, which are already valorized as food or feed (Halim et al., 2016). It is expected that they can equally provide nutrient sources, increase immunity for crops as well. A recent study suggested that FPHs act to promote lettuce growth and stomatal conductance (Xu and Mou, 2017).

Currently, most PHs are from chemically hydrolyzed leather waste (Colantoni et al., 2017). In terms of energy use and environmental impact, animal-derived PHs (e.g., leather by-product) showed higher ecological footprint than plant-derived PHs (Colantoni et al., 2017). As a result, for building sustainable farming, it is preferred to generate biostimulants by enzymatically hydrolyzing plant proteins over animal by-products. By switching to plant-based PHs, it also avoids risks of possible contamination with pathogens.

Several PH biostimulant products have demonstrated activity in enhancing N and C metabolism in plants. For example, a plant-derived PH “Trainer^®^” was used to evaluate the plant growth promoting effects (Colla et al., 2014). The application of Trainer^®^ on maize coleoptiles and dwarf pea induced auxin- and gibberellin-like activities, respectively (Colla et al., 2014). It promotes elongation of maize coleoptiles and shoot length of gibberellin-deficient dwarf pea (Colla et al., 2014). Moreover, it also improves dry weight of shoots and roots, total biomass, cholorphyll content and leaf N content in tomato plants (Colla et al., 2014). The treatment of maize seedlings with one PH derived from hydrolyzed tanning residues resulted in decrease NO^3−^, PO_4_^3−^, and SO_4_^2−^ concentrations (Ertani et al., 2013a). The decreased level of these ions is due to changes of transcripts of genes involved in N metabolism (nitrate reductase, glutamine synthetase, glutamate synthase and asparate aminotransferase) and TCA cycle (malate dehydrogenase, isocitrate dehydrogenase and citrate synthase) (Ertani et al., 2013a).

### Chitin and Chitosan

Chitin, a biopolymer from crustaceans shells, and chitosan, the deacetylated form of chitin, have potential applications in food, cosmetics and industrial processes [Reviewed in (Olsen et al., 2014)]. Chitin and chitosan are co-polymers of N-acetyl-d-glucosamine and d-glucosamine, where the ratio of each monomer in the polymer chain defines its physical, chemical and biological properties (Pichyangkura and Chadchawan, 2015). The binding of chitin and chitosan to cell receptors induces physiological changes, triggering an oxidative burst reaction with H_2_O_2_ accumulation and Ca^2+^ leakage into the cell which are similar to signaling of stress response and in the developmental regulation (du Jardin, 2015). Phenylalanine ammonia-lyase (PAL) is a key plant defense enzyme induced upon contact with chitin molecules, leading to the accumulation of phenolic compounds. Most plant species respond in a similar manner including papaya (*Carica papaya* L.), sweet basil (*Ocimum basilicum* L.), sunflower (*Helianthus annuus* L.), litchi (*Litchi chinensis* Sonn.), grape (*Vitis vinifera* L.), etc. [Reviewed in (Pichyangkura and Chadchawan, 2015)]. The cellular response to chitosan involves also NO, and the phytohormone regulators jasmonic acid (JA), abscisic acid (ABA), and phosphatidic acid (PA), which all relate to abiotic stress gene regulation (Pichyangkura and Chadchawan, 2015). By proteomic approach, a recent study uncovered that enzymes involved in phenylpropanoids biosynthesis in grapes were accumulated in response to chitosan (Lucini et al., 2018).

Due to its potency to induce defense mechanisms and stress response pathways, chitin and chitosan are used to improve crop resilience to pathogen attack and abiotic stress conditions. Chitosan, for example, has been found to be effective against biotrophic and necrotrophic pathogens (Sharp, 2013). Moreover, fungal infection may be affected directly by oligochitosan as the endomembrane system of *Phytophthora capsici* is disrupted, especially the integrity of vacuoles (Xu et al., 2007). The activity can also be indirect, as it involves chitinolytic microbes which form mutualistic relationship with plants by producing chitinase enzymes that degrade chitin-rich tissues from other organism (Sharp, 2013). In terms of yield, preharvest application of chitosan increases fruit yield at harvest (Bautista-Baños et al., 2006). The yield of tomato increases by applying chitosan to soil with *F. oxysporum* f. sp. *radicis-lycopersici* fungal inoculation (Lafontaine and Benhamou, 1996; Bautista-Baños et al., 2006). Chitosan could reduce post-harvesting disease as well, as indicated by several studies showing that chitosan effectively prevents postharvest decay during storage and delays microbial infection [Reviewed in (Bautista-Baños et al., 2006)].

### Other Types of By-Products

Several other types of by-products enhancing plant production and food quality have been reported in recent years. Sugarcane vinasse, a by-product mainly of the sugar-ethanol industry, has been evaluated as a nutrient source for microorganisms to detoxify or remove xenobiotics from the environment, promoting bioremediation of the soil (Christofoletti et al., 2013). Vine shoots generated during the pruning process, have been shown to contain phenolic, volatile and mineral compounds that were applied as grapevine biostimulant and foliar fertilizer (Sánchez-Gómez et al., 2014; Sanchez-Gomez et al., 2017). Aqueous extracts of by-products from fennel, lemon and barley grains were shown to enhance tomato yield and fruit quality (Abou Chehade et al., 2018). Furthermore, it’s not surprising that HS extracted from the by-products of rape, castor oil and flax showed bioactivity in maize plantlet growth, attributed to the phytohormone-like activity (Ertani et al., 2013b).

Aquaponics is an integrated system combining hydroponic crop cultivation with fish cultivation, using the same water for both systems [Reviewed in (Tyson et al., 2011)]. The nutrient by-products generated by the aquaculture could have potential biostimulatory properties, although relative reports are scarce and there’s no solid evidence to substantiate the biostimulatory effects. It is speculated that boosted nutrient uptake of crops was connected with biostimulant elements in the aquaponic water supplemented with macro-nutrients (Nicoletto et al., 2018). It might be due to microorganisms and organic matter dissolved in water that are responsible for the biostimulatory effects (Delaide et al., 2016).

## The Use of Biostimulants Complements Conventional Fertilizers

The increasing use of organic fertilizers is driven by the awareness to quest for organic food industry, replacing conventional farming methods relying heavily on chemical fertilizers. In terms of yield, organic farming performs differently with conventional farming. A 21-year study of agronomic and ecological performance of organic and conventional farming systems revealed that legume-based crop rotations to fertilize the soil organically reduces the input of fertilizer at an expense of 20% lower crop yield compared to conventional fertilization systems (Mader et al., 2002). A comprehensive synthesis of scientific literature on organic farming found that the average organic yield is 25% lower than conventional farming (Seufert et al., 2012). However, the performance of organic farming systems varies substantially depending on the contexts and crops cultivated. Organic fruits and oilseed crops, for instance, are most often as profitable as conventional cultivation (Seufert et al., 2012). The organic systems show efficient resource utilization, increased soil fertility and enhanced floral and faunal diversity (Mader et al., 2002; Diacono and Montemurro, 2011). Another study also indicates that the population of *Trichoderma* species, thermophilic microorganism and enteric bacteria were in greater numbers in organic soil amendment (i.e., composted organic waste) (Bulluck et al., 2002). Meanwhile, in other cases, the yield drop was compensated by the application of vermicompost when applied under specific growing conditions (Arancon et al., 2004; Arancon et al., 2005; Gutiérrez-Miceli et al., 2007). It indicates that by using composts as sources of biostimulants, it can increase yield and decrease loss due to infestation. These findings demonstrate that biostimulants or biofertilizers are relevant components of sustainable agriculture and have the potential to tip the balance in favor of organic cultivation methods, in addition to the fact that comparing yield between different systems is highly subject to contexts. As variations in waste being used to generate vermicompost and biostimulants are a complex mixture of substances showing batch differences that may influence performance, studies to optimize the robustness are needed to avoid variability in the effectiveness of biostimulants products.

The fertilization of soil containing microbial biomass is pH dependent, meaning that urea and ammonium input can lower soil pH over time, posing negative effects on beneficial soil microorganism and crop yield (Geisseler and Scow, 2014). Thus, chemical fertilizers should be applied considering the impact on the environment. Biostimulants could play an important role in improved nutrient uptake by the crop, hereby increase yield, which in turn reduces the dependence on traditional fertilizers. In a study of hydroponically grown rocket (*Eruca sativa* Mill.), biostimulant Actiwave^®^ was added to the nutrient solution of plants grown in floating system (Vernieri et al., 2006). The result showed that Actiwave^®^ increased nutrient uptake and nutrient use efficiency, reduced leaf nitrate content and increased chlorophyll and carotenoids contents (Vernieri et al., 2006). GroZyme^®^, a microbial fermentation product, enriched Zn concentration in the phloem and xylem/collenchyma region of the petiole vascular bundle in sunflower (*H. annuus* L.) (Tian et al., 2014). Field trials of GroZyme^®^ have shown the positive growth effects and elevated K translocation and other nutrient elements, possibly by the microbial extracts forming meal complexes that enhance mineral uptake or mobility (Tian et al., 2014). HS extract, HA7 has been found to stimulate plant growth and leaf chlorophyll content by enhancing N, C and S assimilation in rapeseed (*Brassica napus*) (Jannin et al., 2012). Taken together, sustainable food production and security with minimal impact on the environment will require a combination of organic and conventional cropping systems, which take benefit from the fact that biostimulant application improves mineral absorption by modifying plant innate metabolic pathways and compensates for the reduced use of chemical fertilizers. An important notice is however, that yield increase in organic farming usually takes years to be detected (Bulluck et al., 2002; Seufert et al., 2012).

## Conclusion and Perspectives

Under the framework of circular economy, the development of biostimulants from organic waste has important valorization potential. One of the drivers of market development is the companies in research and development contributing to the expanding list of biostimulants, as well as production and formulation processes (du Jardin, 2015). Currently, there have been some studies on biostimulants originated from organic waste streams, including vermicompost, composted urban waste, sewage sludge. PHs, chitin and chitosan represent the group of biostimulants generated from organic waste. However, a broader profile encompassing molecular and physiological impact of biostimulant on targeted plants is needed. By mode of action analysis, novel groups of compounds with biostimulant activities will be discovered from a variety sources of feedstocks.

The European Commission has proposed to revise EU legislation on waste to set clear targets to establish a long-term goal for waste management (European Commission, 2017). It is expected that the prospect of a circular economy require more research and technology development, focusing on bringing up sustainable products onto the market. Biostimulants extracted from waste streams provides a path for research and industrial partners, from lab research, prototyping, to commercialization and benefit the crop growers and consumers. Eventually, it creates incentives for the society to involve in the sustainable development scheme. Higher relevance to growth parameters (e.g., biomass, SPAD index, plant height, and growth index) can lead to better selection of biostimulatory components from source material, as these components (e.g., soluble or insoluble PH) offer better value and development potentials (Massa et al., 2016).

Agricultural nutrient imbalance is substantial with economic development, from inadequate input to maintain soil fertility in sub-Saharan Africa, to excessive surpluses in many areas in the more developed world (Vitousek et al., 2009). As a result, the use of biostimulants, as an element to increase nutrient use efficiency, should also consider the geo-economic differences across different areas around the world. Monitoring tools for the efficacy of biostimulants are needed to adapt to local and temporal use of biostimulants in agriculture and horticulture (du Jardin, 2015). It is expected that, with the trend of replacing chemical fertilizers, biostimulant derived from by-products will be more commonly used if valorization chain is well established and mode of action is further investigated.

## Author Contributions

LX collected the literature mentioned in the manuscript. LX and DG conceptualized and wrote the manuscript.

## Conflict of Interest Statement

The authors declare that the research was conducted in the absence of any commercial or financial relationships that could be construed as a potential conflict of interest.

## Footnotes

1 http://www.biostimulants.eu

2 http://noshan.eu/index.php/en/

3 http://sunnivaproject.eu/

4 http://www.agrimax-project.eu

5 http://www.funguschain.eu

6 http://newfert.org

7 http://www.horticell.ugent.be/project/bio2bio-biological-waste-streams-biostimulants-and-biopesticides
